# Athletic Differences in the Characteristics of the Photoplethysmographic Pulse Shape: Effect of Maximal Oxygen Uptake and Maximal Muscular Voluntary Contraction

**DOI:** 10.1155/2015/752570

**Published:** 2015-02-02

**Authors:** Anran Wang, Lin Yang, Chengyu Liu, Jingxuan Cui, Yao Li, Xingxing Yang, Song Zhang, Dingchang Zheng

**Affiliations:** ^1^College of Life Science and Bioengineering, Beijing University of Technology, Beijing 100124, China; ^2^School of Control Science and Engineering, Shandong University, Jinan 250061, China; ^3^School of Information Science and Engineering, Shandong University, Jinan 250100, China; ^4^Institute of Cellular Medicine, Newcastle University, Newcastle upon Tyne NE2 4HH, UK

## Abstract

This study aimed to investigate the athletic differences in the characteristics of the photoplethysmographic (PPG) pulse shape. 304 athletes were enrolled and divided into three subgroups according to a typical sport classification in terms of the maximal oxygen uptake (MaxO_2__low, MaxO_2__middle and MaxO_2__high groups) or the maximal muscular voluntary contraction (MMVC_low, MMVC_middle, and MMVC_high groups). Finger PPG pulses were digitally recorded and then normalized to derive the pulse area, pulse peak time *T*_*p*_, dicrotic notch time *T*_*n*_, and pulse reflection index (RI). The four parameters were finally compared between the three subgroups categorized by MaxO_2_ or by MMVC. In conclusion, it has been demonstrated by quantifying the characteristics of the PPG pulses in different athletes that MaxO_2_, but not MMVC, had significant effect on the arterial properties.

## 1. Introduction

The measurement of arterial properties is a common screening tool for the assessment of human health [1, 2]. It has been widely accepted that physical exercise could effectively improve the cardiovascular function and peripheral circulation [3, 4]. It is useful to monitor the physiological state of the arteries in athletes. However, current research on the assessment of arterial properties in athletes is very limited, and the published studies mainly focused on single sport type, such as marathon, triathlon, or swimming athletes [5, 6]. Therefore, it would be scientifically useful to have a comprehensive and systematic research study to assess arterial properties from a wide range of athletes.

Many noninvasive methods have been used to assess or quantify the arterial properties. The arterial pulse propagation time or the pulse wave velocity (PWV) [7, 8] is one of the commonly used methods. The stiffer the artery is, the shorter the pulse propagation time is. A cohort of 492 Japanese-Americans study demonstrated that pulse propagation time can predict cardiovascular disease mortality [9]. The importance of measuring pulse propagation time has already been shown under different clinical conditions [10, 11]. However, in practice, pulse propagation time is calculated from the arrival time of the pulse at the periphery from a reference time, and this is often the R peak of the QRS complex of the electrocardiogram (ECG). Unfortunately, this measurement of pulse propagation time includes the left ventricular electrical depolarization and mechanical preejection times. It cannot precisely represent the properties of the peripheral arteries [12].

Analysis of the characteristics of the arterial pulse shape has also been commonly used. This method only requires the measurement of arterial pulses, and it has been reported that the arterial pulse waveforms are affected by aging [13], heart failure [14], and other physiological or pathological conditions [15–17]. However, there is no existing study to compare the waveform difference of the arterial pulses recorded from different types of athletes.

This study aimed to investigate the athletic differences in the characteristics of the arterial pulse shape. The noninvasive photoplethysmographic (PPG) pulses were used in this study due to its simplicity [18, 19].

## 2. Materials and Methods

### 2.1. Subjects

**Figure 1:**
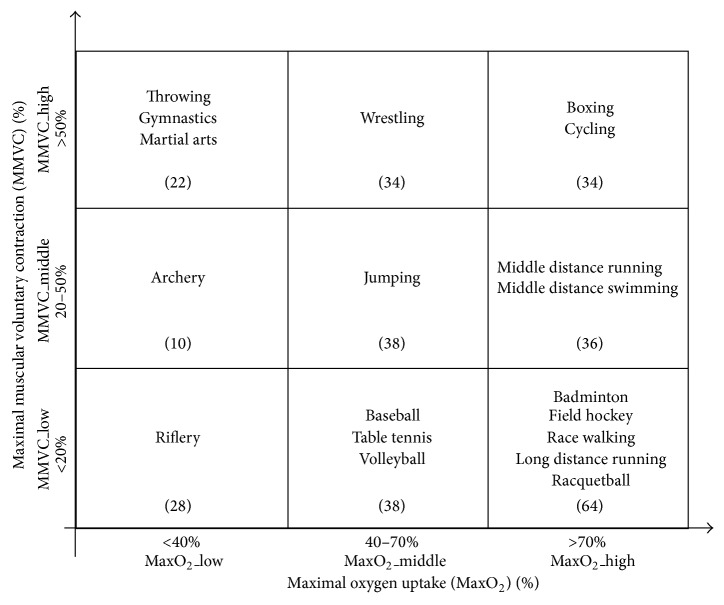
Classification of sports according to dynamic and static exercise components [21]. A total of 304 athletes were studied, and the numbers of athletes in each category are shown.

304 professional athletes were enrolled from Beijing Muxiyuan Sports Technical School. They have been actively trained for at least 3 years, and a wide range of different sports was covered in this study, as shown in Figure 1. This study was fully approved by the local Ethics Committee, College of Life Science and Bioengineering, Beijing University of Technology. Information consent was obtained from each subject prior to the measurement.

A typical sport classification scheme considered that each sport has dynamic and static exercise components [20, 21]. The dynamic exercise component is defined in terms of the percent of maximal oxygen uptake (MaxO_2_) achieved (three levels):

- MaxO_2__low group: MaxO_2_ < 40%;
- MaxO_2__middle group: 40% ≤ MaxO_2_ ≤ 70%;
- MaxO_2__high group: MaxO_2_ > 70%.

The static exercise component is related to the percent of maximal muscular voluntary contraction (MMVC) reached (three levels):

- MMVC_low group: MMVC < 20%;
- MMVC_middle group: 20% ≤  MMVC ≤50%;
- MMVC_high group: MMVC > 50%.

According to the sports classification scheme, all 304 athletes in this study were categorized by the levels of MaxO_2_ (low, middle, and high) and MMVC (low, middle, and high). Figure 1 also shows the number of athletes in each category, and Table 1 gives the overall basic clinical information, including the age, height, and weight, for the three subgroups classified by MaxO_2_ or by MMVC.

### 2.2. PPG Pulse Collection

Each athlete was asked to sit quietly for 3 minutes before the pulse recording and keep relaxing during the whole measurement process. The optical PPG pulses were digitally recorded from the right middle finger using a PowerLab data collection system (ADInstruments Pty Ltd., PowerLab 8/35, Bella Vista, NSW, Australia) at a sampling rate of 1000 Hz. Since there is significant effect of the finger temperature on the PPG pulse shape [22], finger skin temperature was ensured to be around 25°C for each athlete. When the PPG pulse waveforms were satisfactory and stably shown on the monitoring screen, they were saved for 30 s to a computer for further off-line analysis.

### 2.3. Pulse Characteristics Determination

#### 2.3.1. Pulse Waveform Normalization

**Figure 2:**
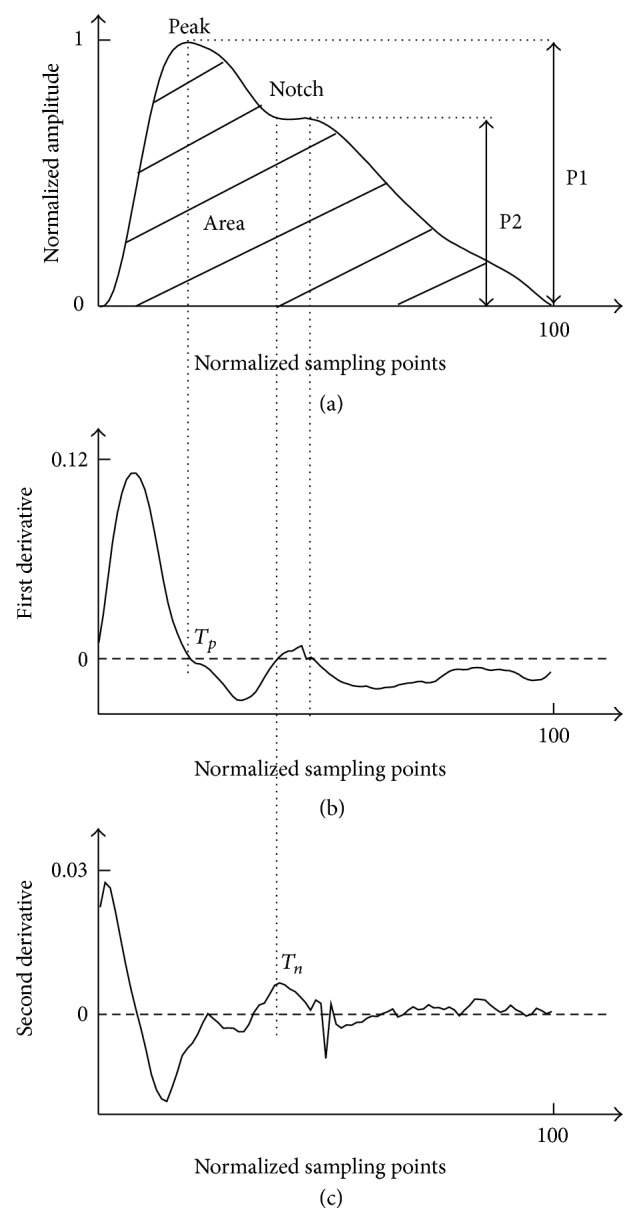
Determination methods of the normalized PPG pulse waveform characteristic. Four parameters were defined, including the pulse area, pulse peak time *T*_*p*_, dicrotic notch time *T*_*n*_, and reflection index RI.

For each athlete, the recorded finger PPG pulses were normalized beat-by-beat in both width (100 sampling points) and amplitude (0-1) from the foot of each pulse and then averaged to obtain a single reference pulse, as shown in Figure 2(a). This normalized pulse was used for subsequent determination of pulse characteristics.

#### 2.3.2. Pulse Area

The “pulse area” describes the global pulse wave characteristics [23], which was computed from the normalized pulse waveform as: Area = ∫_0_^100^ *Y*(*t*)*dt*, as shown in Figure 2(a).

#### 2.3.3. Pulse Peak Time

The pulse peak point *T*_*p*_ was determined from the first derivative PPG pulse, which was corresponding to the first zero-crossing point after the pulse peak, as shown in Figure 2(b).

#### 2.3.4. Dicrotic Notch Time

The pulse dicrotic notch time *T*_*n*_ was determined from the second derivative PPG pulse, which was corresponding to the maximum point after the pulse peak point *T*_*p*_, as shown in Figure 2(c).

#### 2.3.5. Reflection Index (RI)

The PPG pulse has a diastolic peak after the systolic peak [24], and RI measures the vascular resistance of the peripheral arteries. As shown in Figure 2(a), RI was calculated from the height of the diastolic peak (P2) of the PPG waveform as a percentage of the systolic peak (P1), which is expressed as:  RI = P2/P1.

## 3. Results

### 3.1. Pulse Characteristics Difference between Three MaxO_2_ Subgroups

**Figure 3:**
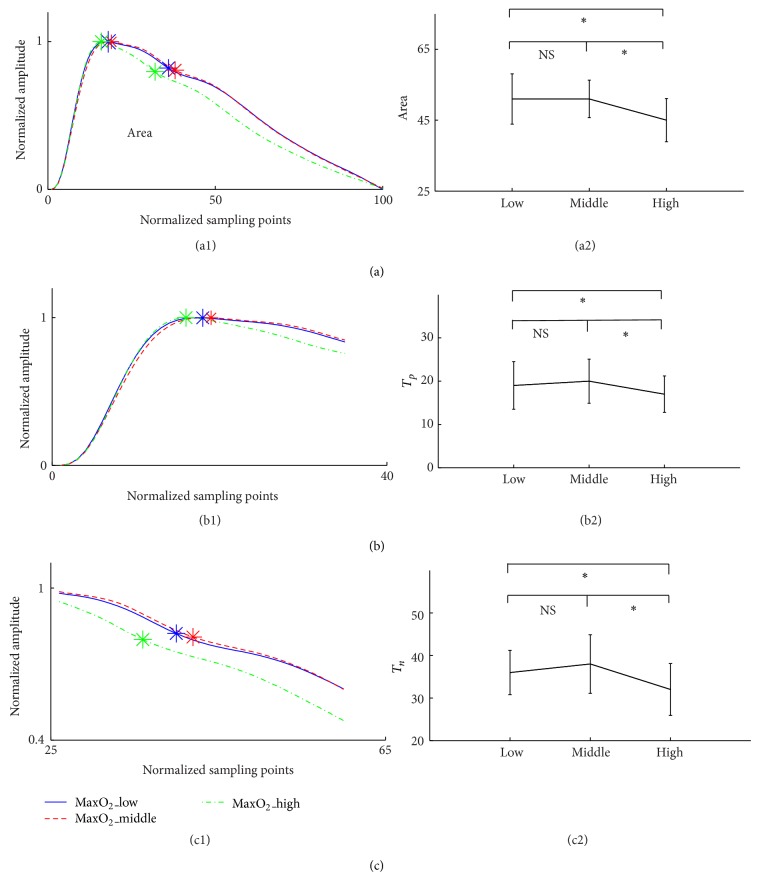
Differences of the PPG pulse characteristics (pulse Area, pulse peak time *T*_*p*_, and dicrotic notch time *T*_*n*_) between the three MaxO_2_ subgroups.

As shown in Figure 3 and Table 2, there were no significant differences in the pulse area, *T*_*p*_, *T*_*n*_, and RI between the MaxO_2__low and MaxO_2__middle groups, but they were all significantly lower in the MaxO_2__high group than both the MaxO_2__low and MaxO_2__middle groups (all *P* < 0.05).

### 3.2. Pulse Characteristics Difference between Three MMVC Subgroups

**Figure 4:**
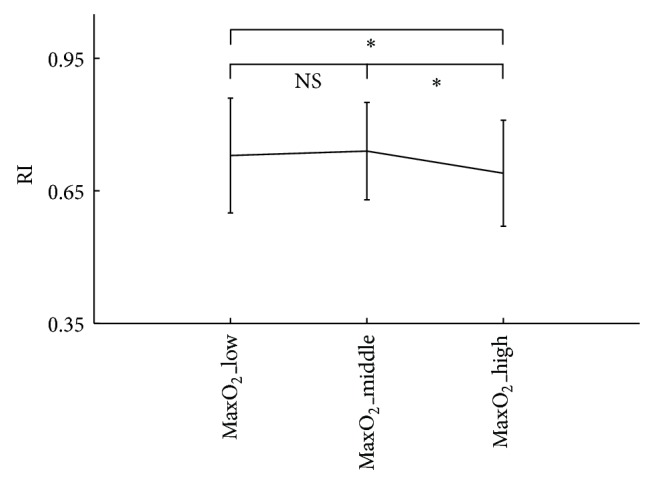
Reflection index (RI) difference between the three MaxO_2_ subgroups.

As shown in Figure 4 and Table 2, there were no significant differences in all pulse characteristics between the three subgroups (all *P* > 0.3), except for the pulse area between the MMVC_low and MMVC_high groups (*P* < 0.05).

## 4. Discussion

The measurement of arterial PPG pulse is an effective method to monitor and evaluate the peripheral artery properties. In this study, the finger PPG pulse shape and their characteristics in different athletes have been quantitatively compared. To the best of our knowledge, this is the first comprehensive study to investigate the athletic effect on arterial pulse shape.

In the athletes requiring high MaxO_2_, the smaller pulse area has been observed in this study. Pulse area has been reported to be increased with the elevation of blood pressure and the increase of arteriosclerosis degree [25]; hence it decreased in subjects with better arterial compliance. The smaller pulse area observed in the MaxO_2__high group indicated that more compliant arteries would be expected in these athletes. Our finding agrees well with some published studies [26–28], where the endurance exercise training (such as long- or middle-distance runners who require higher MaxO_2_) could induce an increased arterial distensibility, whereas the strength exercise training (such as discus, hammer, or javelin throwers) decreased the arterial distensibility.

This study also showed that the pulse peak time, dicrotic notch time, and RI were significantly decreased in the MaxO_2__high group. It has been accepted that, for the younger group with good vascular wall elasticity, the rising velocity of pulse ascending curve is faster or the pulse peak time is shorter when compared with the older group [24]. In addition, since the RI measures peripheral resistance, it has also been accepted that peripheral resistance in the younger group is lower, leading to lower RI, more obvious dicrotic notch point, and shorter time duration between the foot of the pulse and the notch point [24, 29]. With similar physiological mechanisms, because more compliant arteries are expected in the MaxO_2__high group, shorter pulse peak and dicrotic notch times and lower RI would be observed. This has been confirmed in this study.

**Figure 5:**
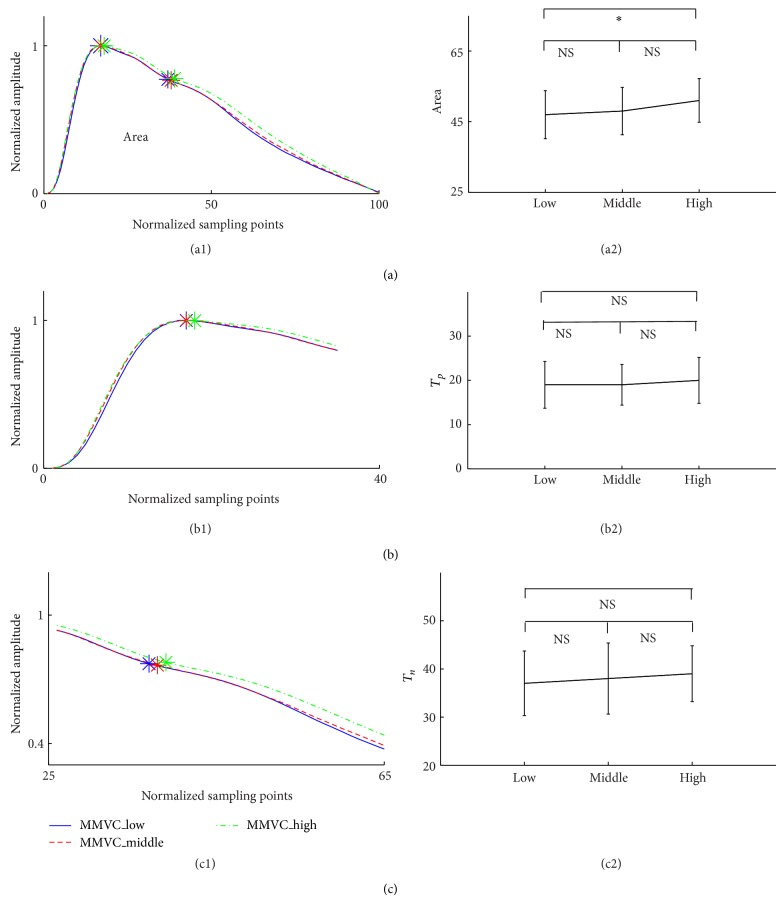
Differences of the PPG pulse characteristics (pulse area, pulse peak time *T*_*p*_, and dicrotic notch time *T*_*n*_) between the three MMVC subgroups.

**Figure 6:**
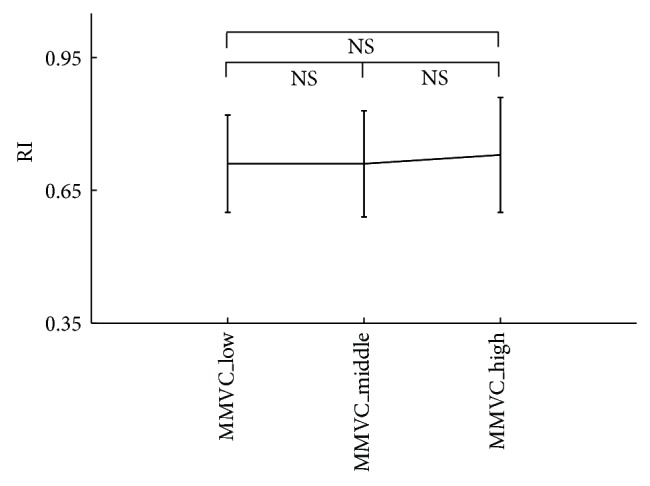
Reflection index (RI) difference between the three MMVC subgroups.

Next, for the effect of MMVC on the arterial properties, it has been published that when compared with the healthy subjects the strength-trained athletes (corresponding to the MMVC_high group in this study) had decreased arterial compliance [28]. Similar result was observed in this study, where smaller pulse area has been quantified in the MMVC_high group in comparison with the MMVC_low group. However, there were no significant differences in the other three pulse characteristics (*T*_*p*_, *T*_*n*_, and RI) between the three MMVC subgroups (Figures 5 and 6), indicating that the effect of MMVC on the decrease of arterial compliance is not so obvious. Further investigations could therefore be followed to better understand the potential difference underlying physiological mechanisms for the different effects of MaxO_2_ and MMVC on arterial properties.

## 5. Conclusion

It has been demonstrated by quantifying the characteristics of the PPG pulses in different athletes that MaxO_2_, but not MMVC, had significant effect on the arterial properties.

## Figures and Tables

**Table 1:** Clinical variables from the 304 athletes studied.

Variables	MaxO_2_ subgroups				MMVC subgroups			
	Low	Middle	High	*P* value	Low	Middle	High	*P* value
No.	60	110	134	0.1	130	84	90	0.4
Gender (F/M)	30/30	55/55	67/67	1.0	65/65	42/42	45/45	1.0
Age, year	20 ± 2	18 ± 4	20 ± 2	<0.05	20 ± 4	20 ± 2	19 ± 3	<0.05
Height, cm	172 ± 10	172 ± 10	174 ± 7	0.3	172 ± 8	177 ± 9	170 ± 9	<0.05
Weight, kg	71 ± 21	63 ± 12	63 ± 9	<0.05	62 ± 12	65 ± 12	67 ± 16	<0.05
HR, beats/min	66 ± 11	66 ± 10	60 ± 10	<0.05	72 ± 9	64 ± 13	66 ± 10	0.1
SBP, mmHg	116 ± 12	118 ± 10	116 ± 9	0.4	116 ± 9	119 ± 10	117 ± 13	0.1
DBP, mmHg	71 ± 9	67 ± 8	72 ± 8	<0.05	72 ± 7	71 ± 8	67 ± 10	<0.05
PP, mmHg	45 ± 10	50 ± 11	45 ± 8	<0.05	44 ± 7	48 ± 9	50 ± 13	<0.05

Note: HR: heart rate, SBP: systolic blood pressure, DBP: diastolic blood pressure, and PP: pulse pressure; *P* value obtained from ANOVA analysis measures the differences between the three subgroups categorized by MaxO_2_ or MMVC.

**Table 2:** Differences of PPG pulse characteristics between the three subgroups catergoised by MaxO_2_ or MMVC.

Parameters	MaxO_2_ subgroups				MMVC subgroups			
	Low	Middle	High	*P* value	Low	Middle	High	*P* value
Area	51 ± 7.1	51 ± 5.3	45 ± 6.1	<0.05	47 ± 6.8	48 ± 6.7	51 ± 6.2	<0.05
*T*_*p*_	19 ± 5.5	20 ± 5.1	17 ± 4.2	<0.05	19 ± 5.3	19 ± 4.6	20 ± 5.2	0.3
*T*_*n*_	36 ± 5.2	38 ± 6.9	32 ± 6.1	<0.05	37 ± 6.7	38 ± 7.4	39 ± 5.8	0.9
RI	0.73 ± 0.13	0.74 ± 0.11	0.69 ± 0.12	<0.05	0.71 ± 0.11	0.71 ± 0.12	0.73 ± 0.13	0.5
